# Drought severity and all-cause mortality rates among adults in the United States: 1968–2014

**DOI:** 10.1186/s12940-020-00597-8

**Published:** 2020-05-18

**Authors:** Katie M. Lynch, Robert H. Lyles, Lance A. Waller, Azar M. Abadi, Jesse E. Bell, Matthew O. Gribble

**Affiliations:** 1 Gangarosa Department of Environmental Health, Emory University Rollins School of Public Health, Atlanta, GA USA; 2 Department of Biostatistics and Bioinformatics, Emory University Rollins School of Public Health, Atlanta, GA USA; 3 Department of Environmental, Agricultural, and Occupational Health, College of Public Health, University of Nebraska Medical Center, Omaha, NE USA; 4 Department of Epidemiology, Emory University Rollins School of Public Health, Atlanta, GA USA

**Keywords:** Climate and health, Global warming, Weather, Droughts, Mortality, Epidemiologic methods

## Abstract

**Background:**

Little is known about the effect of drought on all-cause mortality, especially in higher income countries such as the United States. As the frequency and severity of droughts are likely to increase, understanding the connections between drought and mortality becomes increasingly important.

**Methods:**

Our exposure variable was an annual cumulative drought severity score based on the 1-month, county-level Standardized Precipitation Evapotranspiration Index. The outcome variables of demographic subgroup-specific all-cause mortality count data per year were obtained from the National Vital Statistics System. Any counts below 10 deaths were censored in that demographic group per county. We modeled county-stratum-year mortality using interval-censored negative binomial regression with county-level random intercepts, for each combined age-race-sex stratum either with or without further stratification by climate regions. Fixed effects meta-regression was used to test the associations between age, race, sex, and region with the drought-mortality regression coefficients. Predictive margins were then calculated from the meta-regression model to estimate larger subgroup (e.g., ‘race’ or ‘sex’) associations of drought with mortality.

**Results:**

Most of the results were null for associations between drought severity and mortality, across joint strata of race, age, sex and region, but incidence rate ratios (IRRs) for 17 subgroups were significant after accounting for the multiple testing; ten were < 1 indicating a possible protective effect of drought on mortality for that particular subpopulation. The meta-regression indicated heterogeneity in the association of drought with mortality according to race, climate region, and age, but not by sex. Marginal means of the estimated log-incidence rate ratios differed significantly from zero for age groups 25–34, 35–44, 45–54 and 55–64; for the white race group; and for the South, West and Southwest regions, in the analysis that included wet county-years. The margin of the meta-regression model suggested a slightly negative, but not statistically significant, association of drought with same-year mortality in the overall population.

**Conclusions:**

There were significant, heterogeneous-direction associations in subpopulation-stratified models, after controlling for multiple comparisons, suggesting that the impacts of drought on mortality may not be monolithic across the United States. Meta-regression identified systematic differences in the associations of drought severity with all-cause mortality according to climate region, race, and age. These findings suggest there may be important contextual differences in the effects of drought severity on mortality, motivating further work focused on local mechanisms. We speculate that some of the estimated negative associations of drought severity with same-year mortality could be consistent with either a protective effect of drought on total mortality in the same year, or with a delayed health effect of drought beyond the same year. Further research is needed to clarify associations of drought with more specific causes of death and with sublethal health outcomes, for specific subpopulations, and considering lagged effects occurring beyond the same year as the drought.

## Introduction

Drought generally occurs as a result of a water deficit for a given area, but the actual definition of drought differs depending on the measures considered, such as precipitation and temperature [1]. Droughts are commonly divided into four major types based on their environmental and human impacts, including *meteorological drought* (i.e., abnormally low precipitation), *hydrological drought* (i.e., precipitation shortages that impact the surface and groundwater levels), *agricultural drought* (i.e., decreased soil moisture that impacts crops), and *socioeconomic drought* (i.e., decreased water supply that affects people and supply of goods) [1]. Further, droughts can be characterized by additional factors such as duration, intensity, spatial distribution, frequency, and rate of onset [2].

In 2012, the Intergovernmental Panel on Climate Change (IPCC) reported that medium confidence exists that droughts in some areas of the world have increased in duration and intensity since the 1950s and this pattern will likely continue to over the next century, due to decreased precipitation and/or increased evapotranspiration [3]. A recent IPCC special report found that a shift from 1.5° to 2 °C change in global temperature would cause some areas to experience hotter extreme temperatures, which could increase the probability of dryness and reduced water availability [4]. Within the United States, future climate projections suggest that droughts will be more intense in the Southwest and consecutive dry days are projected to increase over much of the country [5]. At the same time, increases in the heaviest precipitation events are also projected for the U.S., suggesting an increase in both wet and dry extremes could occur [5].

Droughts can lead to widespread and multifaceted regional impacts. Recent droughts in Kenya, the Mediterranean and California have led to crises including food insecurity, political instability, and severe economic damage, respectively [6]. Numerous studies and articles have researched and constructed pathways that connect drought to health effects [7–13]. There are multiple mechanisms by which negative health effects could result from drought. Droughts can cause decreased freshwater availability and impact water quality [13], including a possible increased risk of microbial contamination and cyanobacteria blooms [8, 13, 14]. Other potential pathways through which drought might affect health are through effects on agricultural production and food insecurity [15, 16], increased occurrence and intensity of heat events [6, 17], effects on air quality [18] including through increased risk of wildfires [6, 19, 20] and dust events (which may impact microbial transport and transmission of coccidioidomycosis (Valley fever)) [6, 21–24], altered vector-borne disease transmission dynamics (e.g. West Nile virus) [25, 26], and by possibly acting as a catalyst for negative behavioral or mental health outcomes [27]. Some of these mechanisms can result in disease morbidity and/or mortality [28–44], which may result in certain populations being more at-risk.

A recent systematic review of evidence for the health effects of droughts showed that high-quality, quantitative studies on the association of drought and mortality are limited; while some studies have shown high prevalence of mortality at the time of drought, they do not prove causality [7]. A previous study of all-cause mortality and drought in the United States estimated the percentage of drought-associated health risk in adults aged 65 years and older, comparing full drought, non-drought and worsening drought periods [12]. The study found a 1.55% increased risk of all-cause mortality during high-severity worsening drought periods in western counties of the United States and increased mortality during worsening drought compared to non-drought periods in counties where drought occurred less frequently [12]. A few other studies have examined mortality related to drought among populations outside of the United States. A meta-analysis of aggregated mortality surveys of death rates in Ethiopia concluded there was no likely association between drought and death rates of children under the age of five, but did find an increase in mortality with an increase in the prevalence of global acute malnutrition [9]. A study of suicide risk between 1970 and 2007, among 30–49 year old males in rural New South Wales, Australia, found the relative risk increased 15% (95% confidence interval = 8–22%) when the drought index increased from the 1st to the 3rd quartile, after controlling for season, region and long-term trends; however the risk decreased for rural females over 30 years of age with increasing drought index [11]. Also, a recent study in Spain found statistically significant associations between drought periods and daily mortality in Galicia, north-western Spain [45]. Categorizing the death cases into natural, circulatory and respiratory causes, this article concludes that respiratory cause of mortality was the most strongly associated cause of death [45]. These studies suggest there may be an effect of drought on mortality, but a dearth of studies on the association between drought and mortality in higher income countries remains. In addition, drought health effects could differ by subpopulation, and this is another area in need of more research. Because of this, we chose to evaluate the associations between droughts and same-year all-cause mortality in adults in United States from 1968 to 2014 in the current study.

## Methods

### Data

#### Standardized precipitation evapotranspiration index

Our study’s definition of drought is based on 1-month, county-level Standardized Precipitation Evapotranspiration Index (SPEI) data for the study period of record for the contiguous United States. The SPEI is a climatic drought index based on precipitation and temperature data [46]. Drought indices are quantitative measures derived from the integration of relevant drought indicators (i.e. precipitation) into a single numerical value for drought characterization [2]. Different indices use different variables as indicators, and therefore may reflect different characteristics of drought (e.g. meteorological vs. hydrological). The SPEI is similar to the Standardized Precipitation Index (SPI), but also accounts for the effects of temperature variability on drought assessments through potential evapotranspiration [46]. The SPEI uses meteorological data to determine the summation of deficits and surpluses of available moisture across time, and is based off of historical climatology data for the region [46]. For an SPEI of a given time period, say t months, a time series is constructed by summing the potential evapotranspiration (PET) values from the preceding t-1 months, then fitted to a log-logistic density function to standardize the values (mean 0, SD 1) and make them comparable across time, space, and time scales [47]. For the SPEI used in this analysis, we only used a one-month SPEI. The SPEI ranges from − 3 to 3. Values above 0 indicate relatively wet conditions, and values below 0 indicate relatively dry conditions, with numbers further from zero in either direction representing more extreme conditions [46].

#### Drought severity score

We defined drought to be when two or more contiguous months in a year have each sustained at least at a “moderately dry” category with SPEI ≤ − 1, with the drought accruing a cumulative SPEI ≤ − 5. Each month is associated with a binary indicator of 0 or 1, depending on being part of a drought event or not. We multiplied the indicator for a month being contained in a drought by the SPEI of that month providing a monthly drought score for each county. The annual drought severity score was then calculated by summing the monthly drought scores within each year, for each county. The code for calculating this score is provided in Additional file 1: **Appendix I.**

We also created an analogous wetness severity measure. An abnormally wet period was defined as when 2 or more contiguous months have sustained at least at a “moderately wet” category with SPEI ≥1, with the wet period accruing cumulative SPEI > 5. We multiplied the wet period indicator by the SPEI value (positive during wet period) to obtain a monthly wetness score, which was likewise summed over each year to obtain an annual wetness severity score.

#### All-cause mortality

Mortality data and population counts were extracted from the Mortality Information and Research Analytics (MOIRA) system (formerly known as the Mortality and Population Data System (MPDS) [48, 49], which is a repository and retrieval system for detailed mortality data obtained from the National Center for Health Statistics (NCHS) and the US Census. The database contains death counts and populations within the contiguous United States over 1968–2014, within demographic strata (i.e., age group, race and sex joint categories). The counts for any sub-national cell with fewer than 10 deaths are suppressed per NCHS policy. Our analysis included data from adults ages 25 and older with age categories 25–34, 35–44, 45–54, 55–64, 65–74, 75–84, and 85 years and older.

#### Study sample

This was a complete case analysis that included a total of 5,099,479 observations from 42 demographic strata in 3004 counties and county-equivalents with complete data on stratum-year mortality counts, stratum-year population, and county-year drought scores. Of the observations, 1,610,899 (31.6%) were censored. Of the 6,121,164 observations with mortality counts, county-year-stratum population size was recorded as 0 for 1,013,237 of the observations. A majority of these were from the black and “other” race groups: 596 (0.06%) were from the white race group, 521,091 (51.43%) were from the black race group, and 491,550 (48.51%) were from the “other” race group. Of the observations with population recorded as 0, 1,001,345 (98.85%) had mortality counts recorded as “null” (0). We regarded observations with population equal to 0 as indicating missing data for the actual population size in that county-demographic stratum-year, although it is possible that in some instances a demographic stratum was not represented within a county within a year. These records were therefore excluded from the complete case analysis.

SPEI data was missing for some months or years of some counties, and therefore the calculated drought severity score used in this study was missing. This was the case for 244 county-years (10,248 stratum-county-years). Nine counties were missing SPEI data for all years (0.17%) and were thus excluded entirely from our study. There were 16,380 stratum-county-years without mortality count data. Missing mortality data usually occurred over a consecutive span of several years. For example, La Paz County, Arizona was missing 26 county-years (1092 stratum-county-years) from 1968 to 1993, but had complete data from the remaining years, 1994–2014. Furthermore, La Paz County, Arizona did not form officially until 1983 after separating from Yuma County [50]. This highlights the fact that some of the missing data may be related to changing counties, or counties that formed after 1968, and associated Federal Information Processing System (FIPS) codes for the given spatial location. FIPS codes are unique numerical identifiers for each county or county-equivalent, but the geographic boundaries of counties can change over time, and new counties can form or merge with other counties [50]. As a result, changes to the size and location of a given county in our dataset could occur across time. For example, Yuma County would have decreased in size with the formation of La Paz County.

**Table 1:** Climate regions and included states of the United States

Region	States (Abbreviations)
Central	IL, IN, KY, MO, OH, TN, WV
East North Central	IA, MI, MN, WI
Northeast	CT, DE, ME, MD, MA, NH, NJ, NY, PA, RI, VT, DC
Northwest	ID, OR, WA
South	AR, KS, LA, MS, OK, TX
Southeast	AL, FL, GA, NC, SC, VA
Southwest	AZ, CO, NM, UT
West	CA, NV
West North Central	MT, NE, ND, SD, WY

Overall, out of 6,137,544 potential stratum-county-years that could have been included from the combined datasets, 1,038,065 observations were missing data on drought conditions, mortality, and/or their population had been recorded as 0, resulting in 16.9% of potential county-years being excluded from analysis. However, some of these missing data may be related to changing county boundaries (e.g., counties that formed after 1968), and so spatially those areas may have still been covered even if they appeared to be incomplete records in our dataset. The climate regions, based on those determined by National Oceanic and Atmospheric Association (NOAA), (Table 1) for the United States were used to explore geographic heterogeneity in the association between droughts and mortality [51]. Washington, DC was included with the Northeast climate region because of its geographical location. 

#### Statistical analysis

We modeled county-stratum-year mortality using an interval-censored (ranging from 1 to 9) negative binomial regression model with random intercepts. This model is similar to censored mixed negative binomial regression models used in other studies, except it accounts for interval censoring rather than left or right censoring [52, 53]. One other similar-design epidemiological study used a Bayesian censored mixed-effects Poisson regression model to estimate associations with censored autism counts [54].

The interval censoring accounts for the censored death counts, while the random intercepts allow different county-level baseline mortality rates and account for correlations due to repeated observations of the same county. Our negative binomial regression model assumed a linear predictor for the log of the mean number of deaths of the following form:

$$ \ln \left({\upmu}_{\mathrm{ij}}\right)=\left({\upbeta}_0+{\mathrm{b}}_{0\mathrm{i}}\right)+{\upbeta}_1{\mathrm{X}}_{\mathrm{ij}}+{\upbeta}_2\mathrm{Z}+{\mathrm{f}}_{\mathrm{ij}.} $$

where X_ij_ is the drought score for county i in year j, Z is the potential confounder (year, centered at 1991), and (b_0i_) the random intercept. The offset, f_ij,_ is the ln (population) for each county-stratum-year. When the number of deaths (y_ij_) is 0 or > 10 and not censored, the likelihood contribution for county *i* in year *j*, conditional on the random intercept, is given by

$$ \Pr \left({\mathrm{Y}}_{\mathrm{ij}}={\mathrm{y}}_{\mathrm{ij}}|{\mathrm{X}}_{\mathrm{ij}}={\mathrm{x}}_{\mathrm{ij}},\mathrm{Z}=\mathrm{z}\right)=\frac{\Gamma \left({\upalpha}^{-1}+{\mathrm{y}}_{\mathrm{ij}}\right)}{\Gamma \left({\upalpha}^{-1}\right){\mathrm{y}}_{\mathrm{ij}}!}{\left(\frac{\upalpha {\upmu}_{\mathrm{ij}}}{1+\upalpha {\upmu}_{\mathrm{ij}}}\right)}^{{\mathrm{y}}_{\mathrm{ij}}}{\left(\frac{1}{1+\upalpha {\upmu}_{\mathrm{ij}}}\right)}^{\frac{1}{\upalpha}} $$

where α is the negative binomial dispersion parameter. When the number of deaths is censored over the interval [1, 9], the conditional likelihood contribution, based on the negative binomial model, becomes:

$$ \Pr \left(1\le {\mathrm{Y}}_{\mathrm{ij}}\le 9|{\mathrm{X}}_{\mathrm{ij}}={\mathrm{x}}_{\mathrm{ij}},\mathrm{Z}=\mathrm{z}\right) $$

We implemented maximum likelihood (ML) estimation for this model using the general likelihood facility available in the SAS NLMIXED procedure using SAS 9.3 software (SAS Institute Inc., Cary, NC), which permits user specification of the log-likelihood conditional on the random effects. We specified the contributions for censored observations by taking advantage of recursive properties of the gamma function.

We ran models separately for each of the 42 age-race-sex joint strata. In a second analysis, we further stratified by the nine NOAA climate regions, for a total of 378 strata, to account for potential geographic heterogeneity in the association of drought score with mortality. We repeated the analysis after excluding abnormally wet county-years (wetness severity score > 0) to obtain a comparison of “drought” vs. “normal” years, rather than “drought” vs. “non-drought” years. Forest plots of the results were created using SAS University Edition/SAS 9.4 M6.

The focus for each model was the estimate of β1, i.e., the natural log of the incidence rate ratio (IRR) corresponding to a 1-unit increase in the yearly drought score. Using SAS University Edition/SAS 9.4 M6 (SAS Institute Inc., Cary, NC), we accounted for multiple testing of the ln(IRR)s against the null of 0 using the SAS PROC MULTTEST procedure using the FDR option, which estimates false discovery rate (FDR)-adjusted *p*-values. We adjusted all the p-values from the four sets of analyses: with and without stratification by climate regions, and with and without inclusion of abnormally wet years. We used the estimates from the models that successfully converged. Only models that converged were included in the multiple testing correction as specified in the Results section below.

For both the analyses with and without abnormally wet county-years, we calculated estimated mean deaths for the minimum (0) and maximum (15.01) drought score and the difference between the two, to estimate attributable deaths, for the West U.S. region. We then calculated the total estimated deaths by summing the stratum-specific estimated deaths. We chose the West region because the other regions had models that did not successfully converge. For this region, the drought score was 0 for 96.7% of the observations in the main analysis. We used SAS University Edition/SAS 9.4 M6 (SAS Institute Inc., Cary, NC) for this calculation.

To calculate estimated mean deaths, we exponentiated the right side of the negative binomial equation ((β_0_ + b_0i_) + β_1_ X_ij_ + β_2_ Z + f_ij_)), setting b_0i_ = 0 because the mean and median are 0 by assumption; X_ij_ = 0 (no drought) or the maximum of the drought score for years 1968–2014; Z = 0 (in order to use the mean year, 1991, because we centered the years at 1991); and f_ij_ = ln (mean population for each demographic subgroup across 1968–2014, rounded to nearest integer, among observations without missing values). In calculating the mean population and minimum and maximum drought score, we excluded observations where the population was 0, where the death rate was missing, or where the drought score was missing, because these were not included in the analyses. We rounded estimated deaths to the nearest integer.

We applied fixed effects meta-regression to the age-race-sex-region stratified estimated ln(IRR)s (from analyses with wet years included, or without wet years included) using *vwls* in Stata/SE 14.2. The objective was to assess the association between the regression coefficients from the main analysis (i.e., differences in ln-rates of mortality given drought, within each stratum, adjusted for year and conditional on fixed effects) and potential modifiers of the drought-to-mortality association: age, race, sex, and region. Significance of the association (alpha = 0.05) of each predictor, adjusted for the others, with the drought-mortality regression coefficient was assessed by a Wald test [55].

We then extracted the marginal effects using *margins* in Stata/SE 14.2 for each subgroup and overall. The marginal effects are the adjusted mean value of the estimated ln (IRR) for each demographic and regional subgroup, and overall across all subgroups.

We also completed a logistic regression of missing population with drought severity score as a predictor to check for possible selection bias using SAS University Edition/SAS 9.4 M6 (SAS Institute Inc., Cary, NC).

## Results

Most models in the analyses successfully converged, but there were some estimation problems, where models failed to converge. This issue occurred among the “other” race female subgroup for age categories (years) 25–34, 35–44 and 45–54 in age-race-sex stratified analysis. When stratified by a fourth variable, climate region, 36 of the 378 models failed to converge reliably in the analysis that included abnormally wet county-years. We excluded two additional models that converged, but had estimates suggesting additional convergence issues, i.e. with incident rate ratios (IRRs) of 0.119 (95% confidence interval (CI) = 5.001E− 8, 283,832.31) and 0.14062 (95% CI = 25609E-14, 772,105,431,629). For the analysis stratified by climate regions which excluded abnormally wet county-years, 31 of 378 models failed to converge. We excluded one additional model that resulted in unrealistic IRR of 0.119 (95% CI = 4.797E-8, 297,567.77). The majority of the models that failed to converge were in the “other” race strata.

Overall, most effect estimates were non-significant across categories of race, age, sex, and region, with IRRs for all-cause mortality close to 1. IRRs less than 1 suggest decreasing mortality rates with increasing drought severity, and IRRs greater than 1 suggest increasing mortality rates with increasing drought severity, for a given stratum. When further stratified by climate regions, the majority of results were also null. Exclusion of wet county-years resulted in little to no change in the effect estimates.

A small number of the IRRs were significant after accounting for the multiple testing. These results are presented in Table 2 for the analysis that included abnormally wet county-years, and Additional file 3: **Appendix III,** Table A1 for the sensitivity analysis that excluded abnormally wet county-years. Among the age-race-sex stratified results, from both the analyses with and without inclusion of abnormally wet county-years, there were significant IRRs, all less than 1, for 5 joint strata: white males 25–34, 35–44, 75–84, and 85+ years of age, and white females 25–34 years of age.

**Table 2:** Incidence Rate Ratio (IRR) of all-cause mortality per increasing drought severity by demographic subgroup, with 95% confidence intervals (LCL, UCL), raw *p*-values and false discovery rate-adjusted p-values, for IRRs with adjusted *p* values < 0.05. Abnormally wet years included in analysis

Age	Race	Sex	IRR	LCL	UCL	Raw P	Adjusted P
25–34	White	Male	0.991	0.987	0.995	< 0.0001	0.0006
25–34	White	Female	0.992	0.987	0.997	0.0024	0.0464
35–44	White	Male	0.994	0.991	0.997	< 0.0001	0.0036
75–84	White	Male	0.998	0.997	0.999	0.0001	0.0049
85 +	White	Male	0.998	0.997	0.999	0.0004	0.0116

When further stratified by climate regions, there were 17 significant IRRs from the analysis that included wet county-years (Table 3), and 18 from the analysis that excluded wet county-years (Additional file 3: **Appendix III,** Table A2**)**. Most of these significant estimates were seen in the white, male subgroups, across a range of ages and regions.

**Table 3:** Incidence Rate Ratio (IRR) of all-cause mortality per increasing drought severity by demographic and climate region subgroup, with 95% confidence intervals (LCL, UCL), raw *p*-values and false discovery rate adjusted p-values, for IRRs with adjusted *p* values < 0.05. Abnormally wet years included in analysis

Age	Race	Sex	Region	IRR	LCL	UCL	Raw P	Adjusted P
25–34	White	Male	South	0.987	0.980	0.993	< 0.0001	0.0038
35–44	White	Male	South	0.990	0.985	0.995	0.0001	0.0049
45–54	White	Male	Central	1.011	1.004	1.018	0.0011	0.0253
45–54	White	Male	Southeast	0.990	0.984	0.996	0.0008	0.0196
55–64	White	Male	Northeast	0.991	0.987	0.996	0.0002	0.0051
55–64	White	Male	Southeast	0.992	0.988	0.996	0.0002	0.0051
65–74	White	Male	South	0.996	0.994	0.998	0.0001	0.0049
65–74	White	Female	Southwest	1.007	1.002	1.011	0.0022	0.0464
65–74	White	Female	West	0.991	0.988	0.995	< 0.0001	0.0019
65–74	Black	Male	East North Central	1.034	1.012	1.057	0.0027	0.0464
75–84	White	Male	South	0.996	0.994	0.998	< 0.0001	0.0035
75–84	White	Female	Southwest	1.005	1.002	1.009	0.0020	0.0429
75–84	White	Female	West	0.995	0.992	0.997	< 0.0001	0.0045
75–84	Black	Male	Northeast	1.015	1.008	1.022	< 0.0001	0.0042
75–84	Black	Female	Northeast	1.012	1.005	1.019	0.0006	0.0160
85+	White	Male	South	0.995	0.993	0.997	< 0.0001	0.0012
85+	Black	Female	Northeast	1.012	1.004	1.019	0.0026	0.0464

For both analyses, ten of the significant IRRs were less than 1. Seven significant IRRs were greater than 1 from the analysis that included abnormally wet county-years, and 8 were greater than 1 from the analysis that excluded abnormally wet county-years. The additional significant IRR from the analysis without abnormally wet county-years was for the 65 to 75-year-old “other” race males in the West North Central climate region, with an IRR of 1.066 (95% CI = 1.033, 1.099). This model did not converge in the analysis that included the abnormally wet county-years.

The age-race-sex-region joint strata with IRRs greater than 1 included: 65–74 and 75–84 year-old white females in the Southwest region; 75–84 year-old black males and females, and 85 and older black females in the Northeast region; 45–54 year-old white males in the Central region; and 65–74 year-old black males in the East North Central region. The results for the black and “other” race subgroups usually had wider confidence intervals due to smaller at-risk populations than the white subgroup in some climate regions. Results are also displayed in forest plots in Additional file 4: **Appendix IV**.

In the attributable deaths analysis, for the West climate region, in the analysis with wet county-years included, we estimated that 8837 fewer deaths would have occurred at a drought score of 15.01 (most severe) compared to a drought score of 0 (no drought) in 1991 (Table 4). Excluding the wet county-years, the total estimated attributable deaths for the West climate region was − 8638 (Additional file 3: Table A4). The attributable deaths ranged from − 2092 for 75–84 year old white females to 121 for 65–74 year old black males in the analysis including wet county-years, and from − 2061 for 75–84 year old white females to 124 for 75–84 year old black males for the analysis without wet county-years. Most of the estimated increases in deaths were among minority subpopulations and decreases in deaths were among with white subpopulations.

**Table 4:** Expected same-year deaths in Nevada and California in 1991 for counterfactual scenarios of no drought or most severe drought (from years 1968–2014), and attributable deaths for each age-race-sex stratum. Abnormally wet county-years included in analysis

Age	Race	Sex	Maximum Drought Severity	No Drought	Attributable Deaths
Total			212,746	221,582	−8837
25–34	White	Male	3752	3748	4
25–34	White	Female	1367	1373	−6
25–34	Black	Male	303	334	−31
25–34	Black	Female	226	226	0
25–34	Other	Male	417	387	30
25–34	Other	Female	170	173	−3
35–44	White	Male	4624	5041	−417
35–44	White	Female	2573	2568	4
35–44	Black	Male	506	463	44
35–44	Black	Female	461	424	37
35–44	Other	Male	544	538	6
35–44	Other	Female	337	317	20
45–54	White	Male	8777	9055	−278
45–54	White	Female	5184	5471	−287
45–54	Black	Male	874	876	−2
45–54	Black	Female	700	723	−23
45–54	Other	Male	873	944	−71
45–54	Other	Female	568	583	−15
55–64	White	Male	15,377	15,403	−26
55–64	White	Female	9241	9661	−420
55–64	Black	Male	1440	1408	32
55–64	Black	Female	1154	1061	93
55–64	Other	Male	1384	1272	113
55–64	Other	Female	937	881	56
65–74	White	Male	21,343	22,427	−1084
65–74	White	Female	14,485	16,525	−2040
65–74	Black	Male	1799	1678	121
65–74	Black	Female	1516	1409	107
65–74	Other	Male	1682	1677	5
65–74	Other	Female	1182	1259	−77
75–84	White	Male	26,065	27,114	−1049
75–84	White	Female	24,864	26,956	−2092
75–84	Black	Male	1533	1415	119
75–84	Black	Female	1563	1639	−76
75–84	Other	Male	1770	1899	−129
75–84	Other	Female	1634	1666	−32
85+	White	Male	17,249	17,487	−238
85+	White	Female	29,979	30,958	−979
85+	Black	Male	590	629	−39
85+	Black	Female	1181	1306	−125
85+	Other	Male	1109	1182	−74
85+	Other	Female	1410	1427	−17

The tests for the significance of associations of the categories of race, age, and climate region, with the drought-mortality regression coefficient from the meta-regression, were significant at alpha = 0.05 in the analyses with and without inclusion of wet county-years (Table 5).

**Table 5:** Meta-regression *p* values by covariate, with and without abnormally wet years included in analysis

Covariate	***P*** value
Wet Years Included	
Race	0.001
Sex	0.992
Age	0.002
Region	< 0.001
Wet Years Excluded	
Race	< 0.001
Sex	0.975
Age	0.001
Region	< 0.001

For the marginal effect estimates, besides a couple of differences, the results were mostly similar between the analyses with and without inclusion of wet county-years (Table 6 with wet county-years; Additional file 3: **Appendix III,** Table A3 without wet county-years**)**

**Table 6:** Incident Rate Ratios (IRRs) by demographic and NOAA climate region subgroup and overall across all groups of margins from fixed effects meta-regression controlling for demographic and climate region variables with 95% confidence intervals (LCL, UCL) and *p*-values. Abnormally wet years included in analysis

Subgroup	***P*** value	Estimated Mean IRR	LCL	UCL
**Age Group**				
25–34	0.034	0.997	0.995	1.000
35–44	0.031	0.998	0.996	1.000
45–54	0.029	1.002	1.000	1.003
55–64	0.043	1.001	1.000	1.002
65–74	0.208	1.001	1.000	1.002
75–84	0.657	1.000	0.999	1.001
85+	0.059	1.001	1.000	1.002
**Race**				
White	< 0.001	0.999	0.998	0.999
Black	0.121	1.001	1.000	1.002
Other	0.727	1.000	0.998	1.002
**Sex**				
Male	0.961	1.000	0.999	1.001
Female	0.968	1.000	0.999	1.001
**Region**				
Central	0.731	1.000	0.998	1.001
East North Central	0.567	1.000	0.999	1.002
Northeast	0.213	1.001	1.000	1.002
Northwest	0.796	1.000	0.997	1.002
South	< 0.001	0.997	0.996	0.998
Southeast	0.079	0.999	0.998	1.000
Southwest	< 0.001	1.004	1.003	1.005
West	< 0.001	0.998	0.997	0.999
North West Central	0.220	1.001	0.999	1.004
**Overall**				
	0.962	1.000	0.999	1.001

For the marginal effects, by region, IRRs were significant for the Southwest, South, and West regions for both the analyses with and without wet county-years. The IRR for the Southwest region was greater than 1, while the IRRs for the South and West regions were less than 1. When stratified by race, the IRR for the white subgroup was significant, with a pooled IRR 0.999 (95% CI = 0.998–0.999) for both analyses. The age group stratified meta-regression margins for the analyses that included wet county-years resulted in significant IRRs for 4 age groups: 25–34, 35–44, 45–54, and 55–64 year-olds. The IRRs for the 25–34 and 35–44 year-olds were less than 1, while the IRRs for 45–54 and 55–64 year-olds were greater than 1. Differing from the analysis with wet county-years, results for the 85 year and older age group were significant (IRR > 1), and results for the 25–34 year-old age group were not significant, when wet county-years were excluded. The overall marginal effect estimate without stratification, after adjusting for drought-mortality heterogeneity by demographic and climate variables, was not significant.

## Discussion

The majority of demographic and climate region joint strata showed no significant effect of drought severity on mortality rates within the same year in the contiguous United States from 1968 to 2014. Nonetheless, after adjusting for the false discovery rate, we found significant associations remained for some of the stratified analyses, which differed by subpopulation, suggesting possible health effects for certain groups. Based on the estimated attributable deaths, the net effect of drought severity on mortality was a reduction in deaths in the same year. However as shown in Table 4, a majority of increases in deaths occurred with minority populations.

The tests for significance of the fixed effects meta-regression suggest a linear relationship may exist between the regression coefficients from the main analyses and race, age, and climate region, but not sex, after adjusting for year and other demographic and region variables. The marginal effect estimates showed significant results for specific subgroups of these covariates. In the analysis that included wet county-years, the results of the marginal effect estimates suggest that, after adjustment, average mortality rates decreased slightly with increasing drought severity for 25–34 and 35–44 year-olds, while mortality rates increased slightly with increasing drought severity for 45–54 and 55–64 year-olds. In the analysis with wet county-years, the results did not reflect an increase in mortality rate with increasing drought severity in any of the age groups 65 years or older, but when wet county-years were excluded, the results suggested an increase in mortality with increasing drought severity for the 85 year and older age group. The significant IRRs less than 1 for the 25–34 and 35–44 year-olds could suggest that drought is protective against all-cause mortality in these age strata. If there exist both protective and harmful effects of drought on mortality rates, the protective effects, such as potentially decreased flooding, may drive the IRR below 1 for age groups more likely to be exposed to such events, even though harmful effects might still occur. Older populations may be especially vulnerable to a broader range of the potential effects of drought, such as changes in air quality and heat waves [30, 56], and exposures might differ by age group based on factors such as level of outdoor activity or occupational status, which could explain differential effects of drought on mortality rates by age.

A possible protective effect of drought severity on mortality rates was also seen with the significant marginal effects estimate for the white race group. Although no significant marginal effect estimates were found for the black and “other” race groups, smaller sample sizes and non-converging models could result in bias. If these groups are more sensitive to the effects of drought, the results could be conservative and towards the null. Heterogeneity by race subgroup could exist because race is related to socioeconomic status, which is in turn associated with health disparities [57]. Even after controlling for socioeconomic status, disparities exist which may be attributable to factors such as psychosocial stressors, for example [57].

Finally, for the climate regions, the significant marginal effects estimate for the Southwest region (IRR > 1) suggests average increasing mortality rates with increasing drought severity, while the significant marginal effect estimates for the South and West regions (IRRs < 1) suggest average decreasing mortality rates with increasing drought severity. Particulate matter has been shown to increase in the Southwest during droughts, with one study projecting a 20–130% increase in premature attributable deaths due to particulate matter in this region under a number of climate change projections [58]. This may be a potential mechanism for increased mortality rates with increased drought severity, although this mechanism could exist in other climate regions as well [18]. The results of the West and South regions were unexpected, e.g., because harmful mechanisms such as wildfires in the West are expected to exist, and further research would be needed to see if the results could be replicated.

Overall, based on these results, to the extent to which heterogeneity within different combined demographic groups is negligible, there seems to be a region, age and race effect. Since the marginal effect estimates represent an average of the effects across the joint age-race-sex-region strata from the analyses, certain results could be driving the significance of the groups in the meta-regression, and heterogeneity may still exist. This possible heterogeneity is supported by the significant IRRs found for certain subpopulations.

Regarding age, heterogeneity of effects is suggested by the significant results for some of the age-race-sex-region stratified estimates. Within the 25–34 and 35–44 year-old age groups, results suggested decreasing mortality with increasing drought severity for white males in the South, however there were no other significant race-sex-region subgroups. Among 65–74 year-olds, the IRR was greater than 1 for white females in the Southwest, but the remaining significant stratum-specific IRRs for this age group were less than 1. This heterogeneity within age groups is further supported by IRRs both greater than 1 and less than 1 for strata of age groups 75–84 and 85 years and older.

Among race, heterogeneity was also seen. The white race subgroup had the greatest number of significant IRRs after adjusting for multiple testing. The majority of these significant IRRs suggested decreasing mortality rates with increasing drought severity, across a range of age, sex, and climate region subgroups. One possible reason for the greatest proportion of significant findings from the white race subgroup is that this subgroup frequently had the largest sample size, and more precise estimates. The fact that all significant IRRs for the black race group were greater than 1 could indicate this group is more sensitive to the effects of drought severity on mortality rate, for a given age, sex and region combination. For example, among 75–84 year-old black males and females, and 85 and older black females, in the Northeast, all IRRs were greater than 1. Additionally, the only significant result for “other” race group was also greater than 1 and resulted in the largest effect estimate of all the strata-specific results (IRR:1.066 (95% CI = 1.033, 1.099)); this was for 65–74 year-old males in the West North Central region, in the analysis that excluded abnormally wet years. Although the meta-regression and margins did not indicate sex-specific differences in the average effects of drought severity on mortality rates, there are again certain significant age-race-region subgroups, further supporting heterogeneity by combined strata.

Finally, by region, the results align with the marginal effect estimates in that all the significant results for the Southwest region had IRRs greater than 1 and the South and West regions had IRRs less than 1. These results again suggest heterogeneity because they were only found for certain joint strata. It is possible that any effects of drought severity on mortality rates seen in the Southwest region could differ by exposure and vulnerability among certain demographic groups. There may also be specific health effects related to drought in the Northeast that leave vulnerable populations susceptible to exposure or harmful effects, as seen by the significant results of certain combined age-race-sex strata in this region. The observed heterogeneity indicates differential effects and suggests the effects of drought are contextual, based on specific characteristics of the drought and the vulnerability of the populations. However, we cannot exclude the possibility of false positive results. Further research could determine if the results can be replicated in other settings.

Differences in exposure and vulnerability to climate-related events, potential key determinants of disaster risk and impacts, often leave marginalized groups disproportionately impacted [59]. These can often be attributed to intersecting social processes such as discrimination based on class, gender, or ethnicity, for example, rather than to a single cause, and the effects of the individual’s experiences [59]. Stratifying the analysis by age, race, sex and region may be an oversimplification of this potential intersectionality, because the effects of the individual’s experiences based on these categories may not be additive [60]. Future analyses may examine differences in risk at a local level to examine population and drought heterogeneity on a finer spatial scale. Additionally, other variables not considered in the analysis, such as socioeconomic status or rural vs. urban residence, may be important modifiers to examine in future studies.

Although we did not detect significant associations of drought severity with same-year mortality for most subgroups, it is possible that drought conditions could increase some cause-specific mortality rates while decreasing others, resulting in an overall null result. Our analysis only considered all-cause mortality, and specific types of mortality, such as respiratory mortality or cardiovascular mortality, may still have increased rates due to drought and merit further investigation. If there are cause-specific mortalities that increase due to drought, it is important to understand the causes for targeted interventions. The net effect of a reduction in deaths in the same year could be consistent with a lagged effect of drought severity on mortality beyond the same-year time interval considered in the study.

A recent study of drought and the risk of all-cause mortality among people ages 65 years and older in the western United States found no significant association during full-drought periods or low-severity worsening drought periods, but did find an increased risk of mortality during high-severity worsening drought periods [12]. They also found increased mortality during worsening drought compared to non-drought periods in counties where drought occurred less frequently [12]. This study differed from ours in that it focused on counties west of the Mississippi river only, and was restricted to ages 65 and older [12]. Additionally, the study compared days in drought versus non-drought periods, using the weekly U.S. Drought Monitor, so it used a different index for classifying drought and a different temporal scale [12]. This study further stratified by drought severity, instead of using a continuous variable, and considered factors such as worsening drought, or frequency of droughts in the county, which were not included in our study [12]. For these reasons, the results may not be directly comparable. Considering worsening droughts or frequency of droughts in our models could be an option for further analysis.

A strength of the study was the creation of a novel drought severity score designed to capture drought intensity and duration over the year with a continuous scale from the available one-month SPEI data. This scale allowed us to look for effects related to drought severity, and not simply classify exposure as drought vs. non-drought in the analysis. Another strength of the study is that we developed a way to deal with interval-censored CDC mortality data through an interval-censored negative binomial regression model with random county effects. We then supplemented the subgroup-specific model results by performing the meta-regression and marginal effects analyses. This provides a principled way to incorporate all of the available information about county/month-specific mortality counts, while accounting for the differences between the combined age-race-sex-region strata.

This study has some limitations, including the ecological study design; future analyses might consider alternative designs. There was censoring of the number of deaths over the interval 1 to 9 in the dataset. We used interval censored negative binomial regression to handle the missing data, but this necessarily reduces estimation precision relative to the unavailable complete data, particularly for strata with small numbers at risk. Additionally, there were missing data on population, mortality, and SPEI, further decreasing sample size, and potentially leading to bias in our estimated drought effects if the missing observations differed in terms of drought exposure or mortality from those included. A logistic regression of missing population frequency with drought score as the predictor was completed for the overall dataset, and for the significant strata (Additional file 3: Table A5). Several of the analyses found the drought score estimate significant in the model. As there was an association between exposure and inclusion of a county-year, there is a formal possibility that there could be selection bias if inclusion of a county-year was also differential by the mortality rate in that county-year.

Small strata resulted in non-convergence and wide confidence intervals for some models. This especially affected the “other” race category, which had the greatest number of non-converging models. If the more frequently excluded demographic groups due to smaller sample size are more vulnerable than the included groups, and if they also differ in terms of inclusion based on drought exposure status, selection bias could result for the overall effect estimate, and we may be unable to detect impacts of drought on mortality among particularly vulnerable groups.

Also, despite the strengths of the SPEI, we only used one index for creating the annual drought severity score, whereas another index might have resulted in different classification of drought periods and severity. Additionally, the analysis did not specifically account for differences in the types of droughts, such as agricultural vs. meteorological, which could potentially affect health outcomes differently since they focus on different issues (e.g. effects on crops for agricultural drought) [1]. Further, we did not account for the effect of previous years of drought, except when classifying drought vs. non-drought months for the derivation of the drought severity score. Differences in duration or frequency could theoretically result in different exposure durations to potentially harmful or preventative drought-related conditions or impact a community’s adaptive capacity to droughts.

This analysis did not account for potential lagged effects of drought, or interactions between abnormally wet periods following drought, or drought following abnormally wet periods. Differences could hypothetically exist from mechanisms resulting from increased rainfall after a drought such as landslides, or indirect factors such as changes in water quality [61] and disease transmission (e.g. increased incidence of Valley fever after a period of rain following drought) [7, 62]. Drought and wildfires (which might occur more frequently during droughts) [20] could lead to landslides, in part due to removal of protective vegetation [63], and landslides may be triggered by periods of intense rainfall [63]. Interaction mechanisms could also occur from drought following wet periods. Increased plant growth from a wet period followed by drought might lead to build-up of fuel for wildfires in the form of vegetation, which is related to fire risk and spread [64]. The mechanisms that could impact health related to dry or wet conditions could also differ by geographic location, as suggested by a study that found differences by location (i.e. California and Arizona) in direction of the association between coccidioidomycosis incidence and soil moisture [65]. These types of interactions may be useful avenues for further investigation. Future investigations might also consider a larger suite of potential confounders.

While we also did not directly include a variable for temperature in the analysis, SPEI accounts for temperature when calculating evapotranspiration [46]. Temperature has a complex relationship with drought. Since heat can impact heat-related mortality [37] and influence drought severity through evapotranspiration [47, 66, 67] it could be considered a confounder. Multiple variables are considered in the calculation of evapotranspiration [46] and two droughts of the same SPEI could have different extreme or average high temperatures, and therefore differences in heat-related deaths; however, because heat is a component of drought [46] it may not be logical to separate the two. Heat could also be a mediator on the causal pathway between drought and mortality, since drought conditions can increase heat events [17], which can in turn impact heat-related mortality. Interactions could also possibly occur between the effects of heat and drought on all-cause mortality.

## Conclusions

The lack of significant association between drought severity and all-cause mortality overall, and for many, but not all, subgroups after adjusting for multiple testing, could be, in part, due to the heterogeneity of effects based on categories of race, age, climate region, or other unmeasured variables. Other potential explanations include: contextual heterogeneity in the effects of drought, an observed null effect of subgroups due to cancellation of the protective and harmful effects, effects of drought on cause-specific but not all-cause mortality, limitations in the methods of our analysis, lagged effects of drought on mortality, or some combination of these factors. Our results suggest that the potential associations between drought and all-cause mortality could differ by race, age, and climate region and might be more nuanced than the situations considered here. Therefore, the associations may merit additional research based on more detailed data.

## Supplementary information

**Additional file 1: Appendix I.** Drought Severity Score Code

**Additional file 2: Appendix II.** PROC NLMIXED Code

**Additional file 3: Appendix III.** Sensitivity Analyses

**Additional file 4: Appendix IV.** Forest Plots

## Data Availability

The datasets generated during and/or analyzed during the current study are available in the CDC Vital Statistics Online Data Portal, https://www.cdc.gov/nchs/data_access/vitalstatsonline.htm#Downloadable. The drought/climate datasets during and/or analyzed during the current study are available from the authors on reasonable request.
