# Supplementary material for: Drought severity and all-cause mortality rates among adults in the United States: 1968–2014
Source: Environ Health. 2020 May 18;19:52. doi: 10.1186/s12940-020-00597-8 (PMC7236144; doi:10.1186/s12940-020-00597-8)
Supplement: Supplementary file 1 — Additional file 1: Appendix I. Drought Severity Score Code [file 12940_2020_597_MOESM1_ESM.docx]

Appendix I

Drought Severity Score Code

*Create month lag for SPEI, by county

by fips: gen spei_lag1 = spei[_n-1]

by fips: gen speilag2 = spei[_n-2]

by fips: gen spei_lag3 = spei[_n-3]

by fips: gen spei_lag4 = spei[_n-4]

by fips: gen spei_lead1 = spei[_n + 1]

by fips: gen spei_lead2 = spei[_n + 2]

by fips: gen spei_lead3 = spei[_n + 3]

by fips: gen spei_lead4 = spei[_n + 4]

*The default condition for drought severity is ‘not a drought’.

gen is_drought = 0

*Drop the missing SPEI values from the five counties

drop if spei<− 90

*Berman et al., 2017 [12] defined “is a drought” if 5 continuous months of moderate drought.

replace is_drought = 1 if ((spei_lag4 < = − 1 & spei_lag4! = .) & (spei_lag3 < = − 1 & spei_lag3! = .) & (spei_lag2 < = − 1 & spei_lag2! = .) & (spei_lag1 < = − 1 & spei_lag1! = .) & (spei <= − 1 & spei! = .))

replace is_drought = 1 if ((spei_lag3 < = − 1 & spei_lag3! = .) & (spei_lag2 < = − 1 & spei_lag2! = .) & (spei_lag1 < = − 1 & spei_lag1! = .) & (spei <= − 1 & spei! = .) & (spei_lead1 < = − 1 & spei_lead1! = .))

replace is_drought = 1 if ((spei_lag2 < = − 1 & spei_lag2! = .) & (spei_lag1 < = − 1 & spei_lag1! = .) & (spei <= − 1 & spei! = .) & (spei_lead1 < = − 1 & spei_lead1! = .) & (spei_lead2 < = − 1 & spei_lead2! = .))

replace is_drought = 1 if ((spei_lag1 < = − 1 & spei_lag1! = .) & (spei <= − 1 & spei! = .) & (spei_lead1 < = − 1 & spei_lead1! = .) & (spei_lead2 < = − 1 & spei_lead2! = .) & (spei_lead3 < = − 1 & spei_lead3! = .))

replace is_drought = 1 if ((spei <= − 1 & spei! = .) & (spei_lead1 < = − 1 & spei_lead1! = .) & (spei_lead2 < = − 1 & spei_lead2! = .) & (spei_lead3 < = − 1 & spei_lead3! = .) & (spei_lead4 < = − 1 & spei_lead4! = .))

*At least one of 4 months has an SPEI <= − 2

replace is_drought = 1 if ((spei_lag3 < = − 2 & spei_lag3! = .) & (spei_lag2 < = − 1 & spei_lag2! = .) & (spei_lag1 < = − 1 & spei_lag1! = .) & (spei <= − 1 & spei! = .))

replace is_drought = 1 if ((spei_lag3 < = − 1 & spei_lag3! = .) & (spei_lag2 < = − 2 & spei_lag2! = .) & (spei_lag1 < = − 1 & spei_lag1! = .) & (spei <= − 1 & spei! = .))

replace is_drought = 1 if ((spei_lag3 < = − 1 & spei_lag3! = .) & (spei_lag2 < = − 1 & spei_lag2! = .) & (spei_lag1 < = − 2 & spei_lag1! = .) & (spei <= − 1 & spei! = .))

replace is_drought = 1 if ((spei_lag3 < = − 1 & spei_lag3! = .) & (spei_lag2 < = − 1 & spei_lag2! = .) & (spei_lag1 < = − 1 & spei_lag1! = .) & (spei <= − 2 & spei! = .))

replace is_drought = 1 if ((spei_lag2 < = − 2 & spei_lag2! = .) & (spei_lag1 < = − 1 & spei_lag1! = .) & (spei <= − 1 & spei! = .) & (spei_lead1 < = − 1 & spei_lead1! = .))

replace is_drought = 1 if ((spei_lag2 < = − 1 & spei_lag2! = .) & (spei_lag1 < = − 2 & spei_lag1! = .) & (spei <= − 1 & spei! = .) & (spei_lead1 < = − 1 & spei_lead1! = .))

replace is_drought = 1 if ((spei_lag2 < = − 1 & spei_lag2! = .) & (spei_lag1 < = − 1 & spei_lag1! = .) & (spei <= − 2 & spei! = .) & (spei_lead1 < = − 1 & spei_lead1! = .))

replace is_drought = 1 if ((spei_lag2 < = − 1 & spei_lag2! = .) & (spei_lag1 < = − 1 & spei_lag1! = .) & (spei <= − 1 & spei! = .) & (spei_lead1 < = − 2 & spei_lead1! = .))

replace is_drought = 1 if ((spei_lag1 < = − 2 & spei_lag1! = .) & (spei <= − 1 & spei! = .) & (spei_lead1 < = − 1 & spei_lead1! = .) & (spei_lead2 < = − 1 & spei_lead2! = .))

replace is_drought = 1 if ((spei_lag1 < = − 1 & spei_lag1! = .) & (spei <= − 2 & spei! = .) & (spei_lead1 < = − 1 & spei_lead1! = .) & (spei_lead2 < = − 1 & spei_lead2! = .))

replace is_drought = 1 if ((spei_lag1 < = − 1 & spei_lag1! = .) & (spei <= − 1 & spei! = .) & (spei_lead1 < = − 2 & spei_lead1! = .) & (spei_lead2 < = − 1 & spei_lead2! = .))

replace is_drought = 1 if ((spei_lag1 < = − 1 & spei_lag1! = .) & (spei <= − 1 & spei! = .) & (spei_lead1 < = − 1 & spei_lead1! = .) & (spei_lead2 < = − 2 & spei_lead2! = .))

replace is_drought = 1 if ((spei <= − 2 & spei! = .) & (spei_lead1 < = − 1 & spei_lead1! = .) & (spei_lead2 < = − 1 & spei_lead2! = .) & (spei_lead3 < = − 1 & spei_lead3! = .))

replace is_drought = 1 if ((spei <= − 1 & spei! = .) & (spei_lead1 < = − 2 & spei_lead1! = .) & (spei_lead2 < = − 1 & spei_lead2! = .) & (spei_lead3 < = − 1 & spei_lead3! = .))

replace is_drought = 1 if ((spei <= − 1 & spei! = .) & (spei_lead1 < = − 1 & spei_lead1! = .) & (spei_lead2 < = − 2 & spei_lead2! = .) & (spei_lead3 < = − 1 & spei_lead3! = .))

replace is_drought = 1 if ((spei <= − 1 & spei! = .) & (spei_lead1 < = − 1 & spei_lead1! = .) & (spei_lead2 < = − 1 & spei_lead2! = .) & (spei_lead3 < = − 2 & spei_lead3! = .))

*At least one of 3 months has an SPEI of <= − 3

replace is_drought = 1 if ((spei_lag2 < = − 3 & spei_lag2! = .) & (spei_lag1 < = − 1 & spei_lag1! = .) & (spei <= − 1 & spei! = .))

replace is_drought = 1 if ((spei_lag2 < = − 1 & spei_lag2! = .) & (spei_lag1 < = − 3 & spei_lag1! = .) & (spei <= − 1 & spei! = .))

replace is_drought = 1 if ((spei_lag2 < = − 1 & spei_lag2! = .) & (spei_lag1 < = − 1 & spei_lag1! = .) & (spei <= − 3 & spei! = .))

replace is_drought = 1 if ((spei_lag1 < = − 3 & spei_lag1! = .) & (spei <= − 1 & spei! = .) & (spei_lead1 < = − 1 & spei_lead1! = .))

replace is_drought = 1 if ((spei_lag1 < = − 1 & spei_lag1! = .) & (spei <= − 3 & spei! = .) & (spei_lead1 < = − 1 & spei_lead1! = .))

replace is_drought = 1 if ((spei_lag1 < = − 1 & spei_lag1! = .) & (spei <= − 1 & spei! = .) & (spei_lead1 < = − 3 & spei_lead1! = .))

replace is_drought = 1 if ((spei <= − 3 & spei! = .) & (spei_lead1 < = − 1 & spei_lead1! = .) & (spei_lead2 < = − 1 & spei_lead2! = .))

replace is_drought = 1 if ((spei <= − 1 & spei! = .) & (spei_lead1 < = − 3 & spei_lead1! = .) & (spei_lead2 < = − 1 & spei_lead2! = .))

replace is_drought = 1 if ((spei <= − 1 & spei! = .) & (spei_lead1 < = − 1 & spei_lead1! = .) & (spei_lead2 < = − 3 & spei_lead2! = .))

* Two adjacent months have SPEIs <= − 2 and < = − 3

replace is_drought = 1 if ((spei_lag1 < = − 3 & spei_lag1! = .) & (spei <= − 2 & spei! = .))

replace is_drought = 1 if ((spei_lag1 < = − 2 & spei_lag1! = .) & (spei <= − 3 & spei! = .))

replace is_drought = 1 if ((spei <= − 3 & spei! = .) & (spei_lead1 < = − 2 & spei_lead1! = .))

replace is_drought = 1 if ((spei <= − 2 & spei! = .) & (spei_lead1 < = − 3 & spei_lead1! = .))

* Generate monthly score and sum for each county-year to calculate drought score

gen month_score = is_drought*spei

bysort fips year: egen drought_score = total (month_score)

replace drought_score=. if spei<− 90

gen drought_index = drought_score * -1
