# Supplementary material for: Drought severity and all-cause mortality rates among adults in the United States: 1968–2014
Source: Environ Health. 2020 May 18;19:52. doi: 10.1186/s12940-020-00597-8 (PMC7236144; doi:10.1186/s12940-020-00597-8)
Supplement: Supplementary file 2 — Additional file 2: Appendix II. PROC NLMIXED Code [file 12940_2020_597_MOESM2_ESM.docx]

Appendix II

PROC NLMIXED Code

*Using dataset with drought severity score and mortality data replace suppressed values with number for analysis, delete ages< 25 or population==0, center year at 1991, create offset lnpop, create NOAA regions;

data dat2;

set dat;

if deaths = “SUPPRESSED” then deaths_num = 5;

if population = 0 then delete;

if age_group_13 < 7 then delete;

c_year = year-1991;

lnpop = log (population);

if state = “IL” or state = “IN” or state = “KY” or state = “MO” or state = “OH” or state = “TN” or

state = “WV” then region = 1;

if state = “IA” or state = “MI” or state = “MN” or state = “WI” then region = 2;

if state = “CT” or state = “DE” or state = “ME” or state = “MD” or state = “MA” or state = “NH” or

state = “NJ” or state = “NY” or state = “PA” or state = “RI” or state = “VT” or state = “DC” then

region = 3;

if state = “ID” or state = “OR” or state = “WA” then region = 4;

if state = “AR” or state = “KS” or state = “LA” or state = “MS” or state = “OK” or state = “TX” then

region = 5;

if state = “AL” or state = “FL” or state = “GA” or state = “NC” or state = “SC” or state = “VA” then

region = 6;

if state = “AZ” or state = “CO” or state = “NM” or state = “UT” then region = 7;

if state = “CA” or state = “NV” then region = 8;

if state = “MT” or state = “NE” or state = “ND” or state = “SD” or state = “WY” then region = 9;

if region = 1 then region_label = “CENTRAL”;

if region = 2 then region_label = “EAST_NORTH_CENTRAL”;

if region = 3 then region_label = “NORTH_EAST”;

if region = 4 then region_label = “NORTH_WEST”;

if region = 5 then region_label = “SOUTH”;

if region = 6 then region_label = “SOUTH_EAST”;

if region = 7 then region_label = “SOUTH_WEST”;

if region = 8 then region_label = “WEST”;

if region = 9 then region_label = “WEST_NORTH_CENTRAL”;

*create indicator variable for censoring;

Yobserved = 0;

if deaths_num = 0 | deaths_num ge 10 then Yobserved = 1;

run;

*sort dataset;

proc. sort data = dat2;

by age_group_13 race sex;

run;

**********************************************************************************

Interval Censoring with Random Intercepts Only, Including Wet Years

**********************************************************************************;

*run interval censored negative binomial model with random intercepts INCLUDING wet years, by age/race/sex subgroup;

*use lnpop as the offset and include centered year variable in model;

ODS output ParameterEstimates = parms;

proc. nlmixed data = dat2; ***Interval censored model uses all Y information***;

by age_group_13 race sex;

parms bet0 = 0, bet1 = .5, bet2 = 0, alpha = .2, sigsq1 = .1;

bounds sigsq1 > = 0;

alphainv = 1/alpha;

linp = (bet0 + g0i) + bet1*Drought_Index + bet2*c_year +lnpop;

mu = exp. (linp);

*p* = 1/(1 + mu*alpha);

prYeq0 = p**alphainv;

prYeq1 = alphainv*(p**alphainv)*(1-p);

prYeq2 = ((alphainv+ 1)*alphainv/fact(2))*(p**alphainv)*(1-p)**2;

prYeq3 = ((alphainv+ 2)*(alphainv+ 1)*alphainv/fact(3))*(p**alphainv)*(1-p)**3;

prYeq4 = ((alphainv+ 3)*(alphainv+ 2)*(alphainv+ 1)*alphainv/fact(4))*(p**alphainv)*(1-p)**4;

prYeq5 = ((alphainv+ 4)*(alphainv+ 3)*(alphainv+ 2)*(alphainv+ 1)*alphainv/fact(5))*(p**alphainv)*(1-p)**5;

prYeq6 = ((alphainv+ 5)*(alphainv+ 4)*(alphainv+ 3)*(alphainv+ 2)*(alphainv+ 1)*alphainv/fact(6))*(p**alphainv)*(1-p)**6;

prYeq7 = ((alphainv+ 6)*(alphainv+ 5)*(alphainv+ 4)*(alphainv+ 3)*(alphainv+ 2)*(alphainv+ 1)*alphainv/fact(7))*(p**alphainv)*(1-p)**7;

prYeq8 = ((alphainv+ 7)*(alphainv+ 6)*(alphainv+ 5)*(alphainv+ 4)*(alphainv+ 3)*(alphainv+ 2)*(alphainv+ 1)*alphainv/fact(8))*(p**alphainv)*(1-p)**8;

prYeq9 = ((alphainv+ 8)*(alphainv+ 7)*(alphainv+ 6)*(alphainv+ 5)*(alphainv+ 4)*(alphainv+ 3)*(alphainv+ 2)*(alphainv+ 1)*alphainv/fact(9))*(p**alphainv)*(1-p)**9;

CDFterm = prYeq0 + prYeq1 + prYeq2 + prYeq3 + prYeq4 + prYeq5 + prYeq6 + prYeq7 + prYeq8 + prYeq9;

** log-likelihood function when Y values are detectable ***;

if Yobserved = 1 then do;

loglike = lgamma (alphainv+deaths_num) - lgamma (alphainv) - lgamma(1 + deaths_num)

+ deaths_num*log(1-p) + alphainv*log(p);

end;

** log-likelihood function when Y values are interval censored on [1, 9] ***;

else if Yobserved = 0 then do;

loglike = log (CDFterm - prYeq0);

end;

model deaths_num ~ general (loglike);

random g0i ~ normal(0, sigsq1) subject = fips;

title1 ‘INTERVAL CENSORED (on [1, 9]) NB regression with random effects, using general LL facility’;

title2 ‘Random Intercept Only’;

run;

*save parms to permanent dataset;

data drought.negbin_w_wet;

set parms;

run;

*create permanent dataset with IRRs;

data drought.negbin_w_wetIRR;

set parms;

IRR = exp. (Estimate);

L_IRR = exp. (Lower);

U_IRR = exp. (Upper);

run;

**********************************************************************************

Interval Censoring with Random Intercepts Only by NOAA Region, Including Wet Years

**********************************************************************************;

*sort data;

proc. sort data = dat2;

by age_group_13 race sex region;

run;

*run interval censored negative binomial model with random intercepts INCLUDING wet years, by age/race/sex/region subgroup;

*use lnpop as the offset and include centered year variable in model;

ODS output ParameterEstimates = parms2;

proc. nlmixed data = dat2; ***Interval censored model uses all Y information***;

by age_group_13 race sex region;

parms bet0 = 0, bet1 = .5, bet2 = 0, alpha = .2, sigsq1 = .1;

bounds sigsq1 > = 0;

alphainv = 1/alpha;

linp = (bet0 + g0i) + bet1*Drought_Index + bet2*c_year +lnpop;

mu = exp. (linp);

*p* = 1/(1 + mu*alpha);

prYeq0 = p**alphainv;

prYeq1 = alphainv*(p**alphainv)*(1-p);

prYeq2 = ((alphainv+ 1)*alphainv/fact(2))*(p**alphainv)*(1-p)**2;

prYeq3 = ((alphainv+ 2)*(alphainv+ 1)*alphainv/fact(3))*(p**alphainv)*(1-p)**3;

prYeq4 = ((alphainv+ 3)*(alphainv+ 2)*(alphainv+ 1)*alphainv/fact(4))*(p**alphainv)*(1-p)**4;

prYeq5 = ((alphainv+ 4)*(alphainv+ 3)*(alphainv+ 2)*(alphainv+ 1)*alphainv/fact(5))*(p**alphainv)*(1-p)**5;

prYeq6 = ((alphainv+ 5)*(alphainv+ 4)*(alphainv+ 3)*(alphainv+ 2)*(alphainv+ 1)*alphainv/fact(6))*(p**alphainv)*(1-p)**6;

prYeq7 = ((alphainv+ 6)*(alphainv+ 5)*(alphainv+ 4)*(alphainv+ 3)*(alphainv+ 2)*(alphainv+ 1)*alphainv/fact(7))*(p**alphainv)*(1-p)**7;

prYeq8 = ((alphainv+ 7)*(alphainv+ 6)*(alphainv+ 5)*(alphainv+ 4)*(alphainv+ 3)*(alphainv+ 2)*(alphainv+ 1)*alphainv/fact(8))*(p**alphainv)*(1-p)**8;

prYeq9 = ((alphainv+ 8)*(alphainv+ 7)*(alphainv+ 6)*(alphainv+ 5)*(alphainv+ 4)*(alphainv+ 3)*(alphainv+ 2)*(alphainv+ 1)*alphainv/fact(9))*(p**alphainv)*(1-p)**9;

CDFterm = prYeq0 + prYeq1 + prYeq2 + prYeq3 + prYeq4 + prYeq5 + prYeq6 + prYeq7 + prYeq8 + prYeq9;

** log-likelihood function when Y values are detectable ***;

if Yobserved = 1 then do;

loglike = lgamma (alphainv+deaths_num) - lgamma (alphainv) - lgamma(1 + deaths_num)

+ deaths_num*log(1-p) + alphainv*log(p);

end;

** log-likelihood function when Y values are interval censored on [1, 9] ***;

else if Yobserved = 0 then do;

loglike = log (CDFterm - prYeq0);

end;

model deaths_num ~ general (loglike);

random g0i ~ normal(0, sigsq1) subject = fips;

title1 ‘INTERVAL CENSORED (on [1, 9]) NB regression with random effects, using general LL facility with region’;

title2 ‘Random Intercept Only’;

run;

*save parms2 to permanent dataset;

data drought.negbin_w_wet_NOAA;

set parms2;

run;

*create permanent dataset with IRRs;

data drought.negbin_w_wet_NOAAIRR;

set parms2;

where Parameter = “bet1”;

IRR = exp. (Estimate);

L_IRR = exp. (Lower);

U_IRR = exp. (Upper);

run;

**********************************************************************************

Interval Censoring with Random Intercepts Only, Wet Excluded

*********************************************************************************;

*Create indicator variable for abnormally wet years and remove them from dataset;

data dat3;

set dat2;

if wet_index=. then wet = .;

else if wet_index> 0 then wet = 1;

else wet = 0;

if wet = 1 then delete;

run;

*sort dataset without wet years;

proc. sort data = dat3;

by age_group_13 race sex;

run;

*run interval censored negative binomial model with random intercepts EXCLUDING wet years, by age/race/sex subgroup;

*use lnpop as the offset and include centered year variable in model;

ODS output ParameterEstimates = parms3;

proc. nlmixed data = dat3; ***Interval censored model uses all Y information***;

by age_group_13 race sex;

parms bet0 = 0, bet1 = .5, bet2 = 0, alpha = .2, sigsq1 = .1;

bounds sigsq1 > = 0;

alphainv = 1/alpha;

linp = (bet0 + g0i) + bet1*Drought_Index + bet2*c_year +lnpop;

mu = exp. (linp);

*p* = 1/(1 + mu*alpha);

prYeq0 = p**alphainv;

prYeq1 = alphainv*(p**alphainv)*(1-p);

prYeq2 = ((alphainv+ 1)*alphainv/fact(2))*(p**alphainv)*(1-p)**2;

prYeq3 = ((alphainv+ 2)*(alphainv+ 1)*alphainv/fact(3))*(p**alphainv)*(1-p)**3;

prYeq4 = ((alphainv+ 3)*(alphainv+ 2)*(alphainv+ 1)*alphainv/fact(4))*(p**alphainv)*(1-p)**4;

prYeq5 = ((alphainv+ 4)*(alphainv+ 3)*(alphainv+ 2)*(alphainv+ 1)*alphainv/fact(5))*(p**alphainv)*(1-p)**5;

prYeq6 = ((alphainv+ 5)*(alphainv+ 4)*(alphainv+ 3)*(alphainv+ 2)*(alphainv+ 1)*alphainv/fact(6))*(p**alphainv)*(1-p)**6;

prYeq7 = ((alphainv+ 6)*(alphainv+ 5)*(alphainv+ 4)*(alphainv+ 3)*(alphainv+ 2)*(alphainv+ 1)*alphainv/fact(7))*(p**alphainv)*(1-p)**7;

prYeq8 = ((alphainv+ 7)*(alphainv+ 6)*(alphainv+ 5)*(alphainv+ 4)*(alphainv+ 3)*(alphainv+ 2)*(alphainv+ 1)*alphainv/fact(8))*(p**alphainv)*(1-p)**8;

prYeq9 = ((alphainv+ 8)*(alphainv+ 7)*(alphainv+ 6)*(alphainv+ 5)*(alphainv+ 4)*(alphainv+ 3)*(alphainv+ 2)*(alphainv+ 1)*alphainv/fact(9))*(p**alphainv)*(1-p)**9;

CDFterm = prYeq0 + prYeq1 + prYeq2 + prYeq3 + prYeq4 + prYeq5 + prYeq6 + prYeq7 + prYeq8 + prYeq9;

** log-likelihood function when Y values are detectable ***;

if Yobserved = 1 then do;

loglike = lgamma (alphainv+deaths_num) - lgamma (alphainv) - lgamma(1 + deaths_num)

+ deaths_num*log(1-p) + alphainv*log(p);

end;

** log-likelihood function when Y values are interval censored on [1, 9] ***;

else if Yobserved = 0 then do;

loglike = log (CDFterm - prYeq0);

end;

model deaths_num ~ general (loglike);

random g0i ~ normal(0, sigsq1) subject = fips;

title1 ‘INTERVAL CENSORED (on [1, 9]) NB regression with random effects, using general LL facility’;

title2 ‘Random Intercept Only’;

run;

*save parms3 to permanent dataset;

data drought.negbin_no_wet;

set parms3;

run;

*create permanent dataset with IRRs;

data drought.negbin_no_wetIRR;

set parms3;

where Parameter = “bet1”;

IRR = exp. (Estimate);

L_IRR = exp. (Lower);

U_IRR = exp. (Upper);

run;

*********************************************************************************

Interval Censoring with Random Intercepts Only by NOAA Region, Wet Excluded

********************************************************************************;

*sort dataset without wet years;

proc. sort data = dat3;

by age_group_13 race sex region;

run;

*run interval censored negative binomial model with random intercepts EXCLUDING wet years, by age/race/sex/region subgroup;

*use lnpop as the offset and include centered year variable in model;

ODS output ParameterEstimates = parms4;

proc. nlmixed data = dat3; ***Interval censored model uses all Y information***;

by age_group_13 race sex region;

parms bet0 = 0, bet1 = .5, bet2 = 0, alpha = .2, sigsq1 = .1;

bounds sigsq1 > = 0;

alphainv = 1/alpha;

linp = (bet0 + g0i) + bet1*Drought_Index + bet2*c_year +lnpop;

mu = exp. (linp);

*p* = 1/(1 + mu*alpha);

prYeq0 = p**alphainv;

prYeq1 = alphainv*(p**alphainv)*(1-p);

prYeq2 = ((alphainv+ 1)*alphainv/fact(2))*(p**alphainv)*(1-p)**2;

prYeq3 = ((alphainv+ 2)*(alphainv+ 1)*alphainv/fact(3))*(p**alphainv)*(1-p)**3;

prYeq4 = ((alphainv+ 3)*(alphainv+ 2)*(alphainv+ 1)*alphainv/fact(4))*(p**alphainv)*(1-p)**4;

prYeq5 = ((alphainv+ 4)*(alphainv+ 3)*(alphainv+ 2)*(alphainv+ 1)*alphainv/fact(5))*(p**alphainv)*(1-p)**5;

prYeq6 = ((alphainv+ 5)*(alphainv+ 4)*(alphainv+ 3)*(alphainv+ 2)*(alphainv+ 1)*alphainv/fact(6))*(p**alphainv)*(1-p)**6;

prYeq7 = ((alphainv+ 6)*(alphainv+ 5)*(alphainv+ 4)*(alphainv+ 3)*(alphainv+ 2)*(alphainv+ 1)*alphainv/fact(7))*(p**alphainv)*(1-p)**7;

prYeq8 = ((alphainv+ 7)*(alphainv+ 6)*(alphainv+ 5)*(alphainv+ 4)*(alphainv+ 3)*(alphainv+ 2)*(alphainv+ 1)*alphainv/fact(8))*(p**alphainv)*(1-p)**8;

prYeq9 = ((alphainv+ 8)*(alphainv+ 7)*(alphainv+ 6)*(alphainv+ 5)*(alphainv+ 4)*(alphainv+ 3)*(alphainv+ 2)*(alphainv+ 1)*alphainv/fact(9))*(p**alphainv)*(1-p)**9;

CDFterm = prYeq0 + prYeq1 + prYeq2 + prYeq3 + prYeq4 + prYeq5 + prYeq6 + prYeq7 + prYeq8 + prYeq9;

** log-likelihood function when Y values are detectable ***;

if Yobserved = 1 then do;

loglike = lgamma (alphainv+deaths_num) - lgamma (alphainv) - lgamma(1 + deaths_num)

+ deaths_num*log(1-p) + alphainv*log(p);

end;

** log-likelihood function when Y values are interval censored on [1, 9] ***;

else if Yobserved = 0 then do;

loglike = log (CDFterm - prYeq0);

end;

model deaths_num ~ general (loglike);

random g0i ~ normal(0, sigsq1) subject = fips;

title1 ‘INTERVAL CENSORED (on [1, 9]) NB regression with random effects by Region, using general LL facility with region’;

title2 ‘Random Intercept Only’;

run;

*save parms4 to permanent dataset;

data drought.negbin_no_wetNOAA;

set parms4;

run;

*Create permanent dataset of IRRs;

data drought.negbin_no_wetNOAAIRR;

set parms4;

where Parameter = “bet1”;

IRR = exp. (Estimate);

L_IRR = exp. (Lower);

U_IRR = exp. (Upper);

run;
