# Supplementary material for: Drought severity and all-cause mortality rates among adults in the United States: 1968–2014
Source: Environ Health. 2020 May 18;19:52. doi: 10.1186/s12940-020-00597-8 (PMC7236144; doi:10.1186/s12940-020-00597-8)
Supplement: Supplementary file 3 — Additional file 3: Appendix III. Sensitivity Analyses [file 12940_2020_597_MOESM3_ESM.docx]

Appendix III

Sensitivity Analyses

Table A1 Incidence Rate Ratio (IRR) of all-cause mortality per increasing drought severity by demographic subgroup, with 95% confidence intervals (LCL, UCL), raw *p*-values and false discovery rate adjusted p-values, for IRRs with adjusted *p* values < 0.05. Abnormally wet years excluded from analysis

| **Age** | **Race** | **Sex** | **IRR** | **LCL** | **UCL** | **Raw P** | **Adjusted P** |
| --- | --- | --- | --- | --- | --- | --- | --- |
| 25–34 | White | Male | 0.991 | 0.987 | 0.995 | < 0.0001 | 0.0006 |
| 25–34 | White | Female | 0.992 | 0.987 | 0.997 | 0.0027 | 0.0464 |
| 35–44 | White | Male | 0.994 | 0.991 | 0.997 | < 0.0001 | 0.0035 |
| 75–84 | White | Male | 0.998 | 0.997 | 0.999 | 0.0002 | 0.0051 |
| 85 + | White | Male | 0.998 | 0.997 | 0.999 | 0.0004 | 0.0116 |

Table A2 Incidence Rate Ratio (IRR) of all-cause mortality per increasing drought severity by demographic and climate region subgroup, with 95% confidence intervals (LCL, UCL), raw p-values and false discovery rate adjusted, for IRRs with adjusted p-values < 0.05. Abnormally wet years excluded from analysis

| **Age** | **Race** | **Sex** | **Region** | **IRR** | **LCL** | **UCL** | **Raw P** | **Adjusted P** |
| --- | --- | --- | --- | --- | --- | --- | --- | --- |
| 25–34 | White | Male | South | 0.987 | 0.981 | 0.993 | < 0.0001 | 0.0044 |
| 35–44 | White | Male | South | 0.990 | 0.985 | 0.995 | 0.0002 | 0.0051 |
| 45–54 | White | Male | Central | 1.011 | 1.004 | 1.018 | 0.0012 | 0.0269 |
| 45–54 | White | Male | Southeast | 0.990 | 0.984 | 0.996 | 0.0011 | 0.0253 |
| 55–64 | White | Male | Northeast | 0.991 | 0.987 | 0.996 | 0.0002 | 0.0057 |
| 55–64 | White | Male | Southeast | 0.992 | 0.988 | 0.996 | 0.0002 | 0.0062 |
| 65–74 | White | Male | South | 0.996 | 0.994 | 0.998 | 0.0001 | 0.0049 |
| 65–74 | White | Female | Southwest | 1.007 | 1.002 | 1.011 | 0.0024 | 0.0464 |
| 65–74 | White | Female | West | 0.991 | 0.988 | 0.995 | < 0.0001 | 0.0019 |
| 65–74 | Black | Male | East North Central | 1.034 | 1.012 | 1.057 | 0.0027 | 0.0464 |
| 65–74 | Other | Male | West North Central | 1.066 | 1.033 | 1.099 | < 0.0001 | 0.0044 |
| 75–84 | White | Male | South | 0.996 | 0.995 | 0.998 | < 0.0001 | 0.0037 |
| 75–84 | White | Female | Southwest | 1.005 | 1.002 | 1.009 | 0.0025 | 0.0464 |
| 75–84 | White | Female | West | 0.995 | 0.992 | 0.997 | 0.0001 | 0.0049 |
| 75–84 | Black | Male | Northeast | 1.015 | 1.008 | 1.022 | < 0.0001 | 0.0044 |
| 75–84 | Black | Female | Northeast | 1.012 | 1.005 | 1.019 | 0.0007 | 0.0162 |
| 85+ | White | Male | South | 0.995 | 0.993 | 0.997 | < 0.0001 | 0.0012 |
| 85+ | Black | Female | Northeast | 1.012 | 1.004 | 1.019 | 0.0026 | 0.0464 |

Table A3 Incident Rate Ratios (IRRs) by demographic and NOAA climate region subgroup of margins from fixed effects meta-regression controlling for demographic and climate region variables with 95% confidence intervals (LCL, UCL) and p values. Abnormally wet years excluded from analysis. *indicates significant at alpha = 0.05

| **Subgroup** | **P value** | **IRR** | **LCL** | **UCL** |
| --- | --- | --- | --- | --- |
| **Age Group** |  |  |  |  |
| 25–34 | 0.056 | 0.998 | 0.995 | 1.000 |
| 35–44 | 0.037* | 0.998 | 0.996 | 1.000 |
| 45–54 | 0.015* | 1.002 | 1.000 | 1.003 |
| 55–64 | 0.015* | 1.001 | 1.000 | 1.003 |
| 65–74 | 0.118 | 1.001 | 1.000 | 1.002 |
| 75–84 | 0.484 | 1.000 | 0.999 | 1.001 |
| 85+ | 0.033* | 1.001 | 1.000 | 1.002 |
| **Race** | | | | |
| White | 0.000* | 0.999 | 0.998 | 1.000 |
| Black | 0.097 | 1.001 | 1.000 | 1.002 |
| Other | 0.449 | 1.001 | 0.999 | 1.003 |
| **Sex** | | | | |
| Male | 0.749 | 1.000 | 0.999 | 1.001 |
| Female | 0.735 | 1.000 | 0.999 | 1.001 |
| **Region** | | | | |
| Central | 0.841 | 1.000 | 0.999 | 1.001 |
| East North Central | 0.334 | 1.001 | 0.999 | 1.002 |
| Northeast | 0.163 | 1.001 | 1.000 | 1.002 |
| Northwest | 0.929 | 1.000 | 0.997 | 1.002 |
| South | 0.000* | 0.997 | 0.996 | 0.998 |
| Southeast | 0.086 | 0.999 | 0.998 | 1.000 |
| Southwest | 0.000* | 1.004 | 1.003 | 1.005 |
| West | 0.000* | 0.998 | 0.997 | 0.999 |
| North West Central | 0.115 | 1.002 | 1.000 | 1.004 |
| **Overall** | | | | |
| Across Groups | 0.720 | 1.000 | 0.999 | 1.001 |

Table A4 Expected same-year deaths in Nevada and California in 1991 for counterfactual scenarios of no drought or most severe drought (from years 1968–2014), and attributable deaths for each age-race-sex stratum. Abnormally wet county-years excluded from analysis

| **Age** | **Race** | **Sex** | **Maximum Drought Severity** | **No drought** | **Attributable Deaths** |
| --- | --- | --- | --- | --- | --- |
| Total |  |  | 212,963 | 221,601 | −8638 |
| 25–34 | White | Male | 3750 | 3746 | 5 |
| 25–34 | White | Female | 1367 | 1371 | −4 |
| 25–34 | Black | Male | 305 | 336 | −31 |
| 25–34 | Black | Female | 228 | 225 | 2 |
| 25–34 | Other | Male | 417 | 390 | 28 |
| 25–34 | Other | Female | 169 | 173 | −4 |
| 35–44 | White | Male | 4622 | 5040 | − 419 |
| 35–44 | White | Female | 2572 | 2565 | 7 |
| 35–44 | Black | Male | 505 | 463 | 42 |
| 35–44 | Black | Female | 459 | 424 | 35 |
| 35–44 | Other | Male | 548 | 540 | 7 |
| 35–44 | Other | Female | 335 | 316 | 20 |
| 45–54 | White | Male | 8795 | 9058 | − 263 |
| 45–54 | White | Female | 5199 | 5475 | − 276 |
| 45–54 | Black | Male | 879 | 877 | 3 |
| 45–54 | Black | Female | 698 | 721 | −23 |
| 45–54 | Other | Male | 877 | 944 | −67 |
| 45–54 | Other | Female | 569 | 581 | −11 |
| 55–64 | White | Male | 15,387 | 15,404 | −17 |
| 55–64 | White | Female | 9257 | 9660 | −403 |
| 55–64 | Black | Male | 1431 | 1406 | 25 |
| 55–64 | Black | Female | 1154 | 1062 | 92 |
| 55–64 | Other | Male | 1386 | 1273 | 113 |
| 55–64 | Other | Female | 939 | 885 | 54 |
| 65–74 | White | Male | 21,362 | 22,426 | − 1064 |
| 65–74 | White | Female | 14,488 | 16,516 | − 2029 |
| 65–74 | Black | Male | 1799 | 1678 | 121 |
| 65–74 | Black | Female | 1518 | 1413 | 105 |
| 65–74 | Other | Male | 1678 | 1672 | 6 |
| 65–74 | Other | Female | 1185 | 1258 | −73 |
| 75–84 | White | Male | 26,110 | 27,115 | − 1005 |
| 75–84 | White | Female | 24,904 | 26,965 | −2061 |
| 75–84 | Black | Male | 1540 | 1416 | 124 |
| 75–84 | Black | Female | 1567 | 1641 | −73 |
| 75–84 | Other | Male | 1771 | 1898 | − 127 |
| 75–84 | Other | Female | 1637 | 1668 | −31 |
| 85+ | White | Male | 17,256 | 17,484 | − 229 |
| 85+ | White | Female | 30,003 | 30,973 | −970 |
| 85+ | Black | Male | 590 | 627 | −38 |
| 85+ | Black | Female | 1184 | 1307 | − 123 |
| 85+ | Other | Male | 1110 | 1178 | −69 |
| 85+ | Other | Female | 1414 | 1429 | −15 |

Table A5 Demographics, p-value from logistic regression of missing population frequency with drought as predictor, missing population frequency, and missing drought score frequency for significant strata from main analyses.*

| **Age** | **Race** | **Sex** | **Region** | **p value** | **Missing population frequency** | **Missing drought score frequency**  **(not in model)** |
| --- | --- | --- | --- | --- | --- | --- |
| With wet years | | | | | | |
| Overall | | | | <.0001 | 1,011,437 | 10,248 |
| 25–34 | White | Female | N/A | 0.0384 | 7 | 244 |
| 75–84 | White | Male | N/A | 0.1871 | 14 | 244 |
| 85 + | White | Male | N/A | 0.0256 | 254 | 244 |
| 65–74 | White | Male | South | 0.0129 | 1 | 0 |
| 65–74 | Black | Male | East North Central | 0.0122 | 8094 | 0 |
| 75–84 | White | Male | South | 0.388 | 14 | 0 |
| 75–84 | Black | Male | Northeast | 0.0141 | 1610 | 0 |
| 75–84 | Black | Female | Northeast | 0.0061 | 1483 | 0 |
| 85+ | White | Male | South | 0.0812 | 104 | 0 |
| 85+ | Black | Female | Northeast | 0.0010 | 2415 | 0 |
| Without wet years | | | | | | |
| Overall | | | | <.0001 | 1,003,041 | 10,248 |
| 25–34 | White | Female | N/A | 0.0391 | 7 | 244 |
| 75–84 | White | Male | N/A | 0.1893 | 14 | 244 |
| 85 + | White | Male | N/A | 0.0262 | 253 | 244 |
| 65–74 | White | Male | South | 0.0131 | 1 | 0 |
| 65–74 | Black | Male | East North Central | 0.0113 | 8006 | 0 |
| 65–74 | Other | Male | West North Central | 0.8322 | 6054 | 21 |
| 75–84 | White | Male | South | 0.3931 | 14 | 0 |
| 75–84 | Black | Male | Northeast | 0.0133 | 1605 | 0 |
| 75–84 | Black | Female | Northeast | 0.0059 | 1477 | 0 |
| 85+ | White | Male | South | 0.0853 | 104 | 0 |
| 85+ | Black | Female | Northeast | 0.0009 | 2406 | 0 |

*Among observations without missing drought score or death data. Remaining significant strata from main analysis either did not have missing population, or also had missing drought score for observations that were missing population
