# Supplementary figures and images for: Drought severity and all-cause mortality rates among adults in the United States: 1968–2014
Source: Environ Health. 2020 May 18;19:52. doi: 10.1186/s12940-020-00597-8 (PMC7236144; doi:10.1186/s12940-020-00597-8)

Appendix IV

Forest Plots


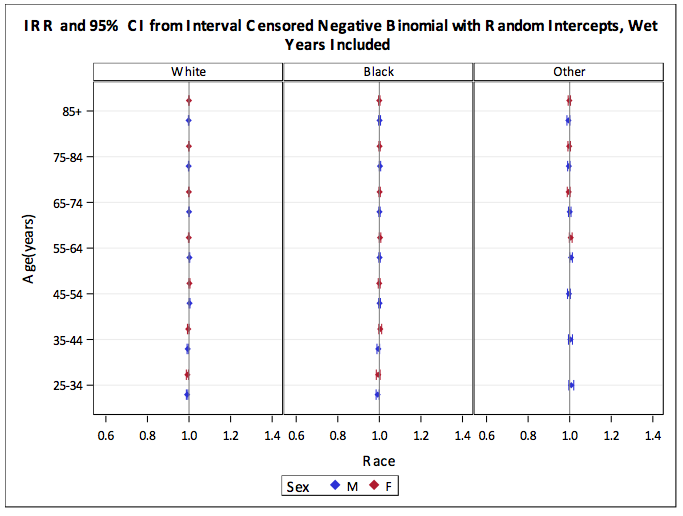


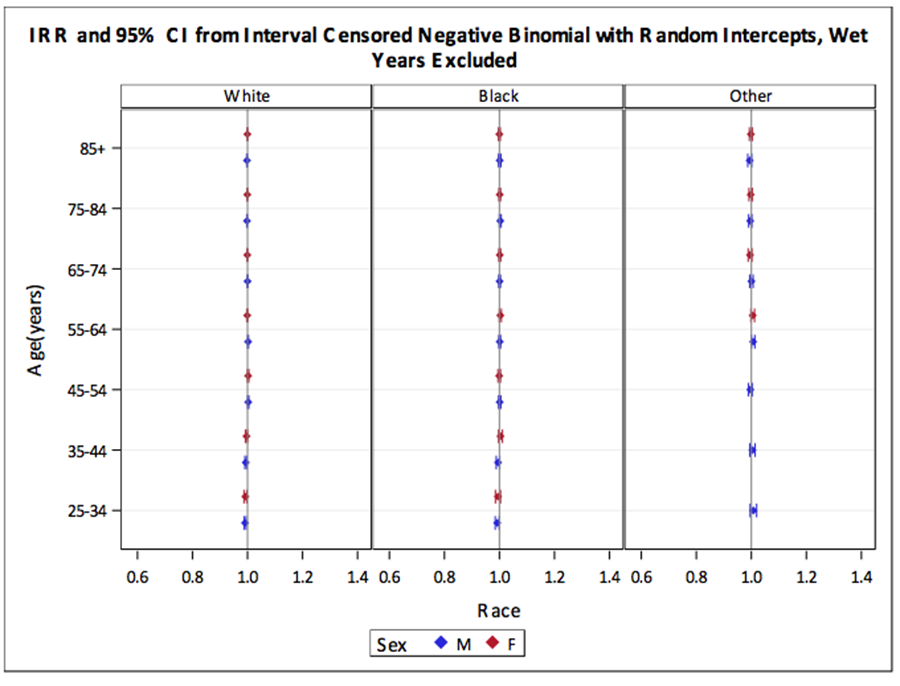


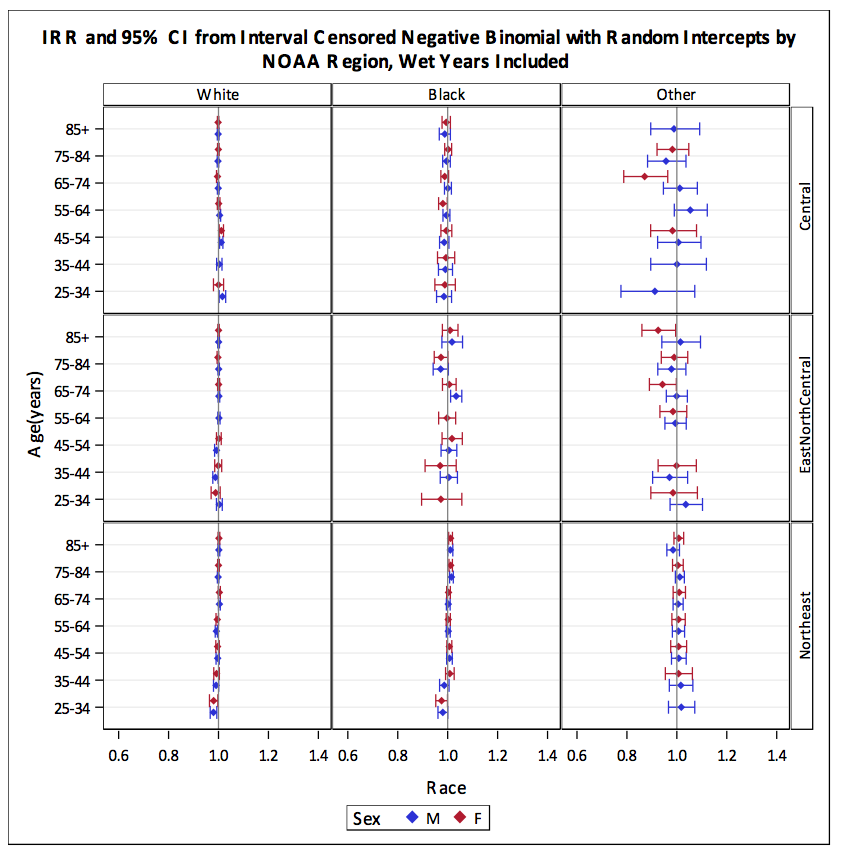


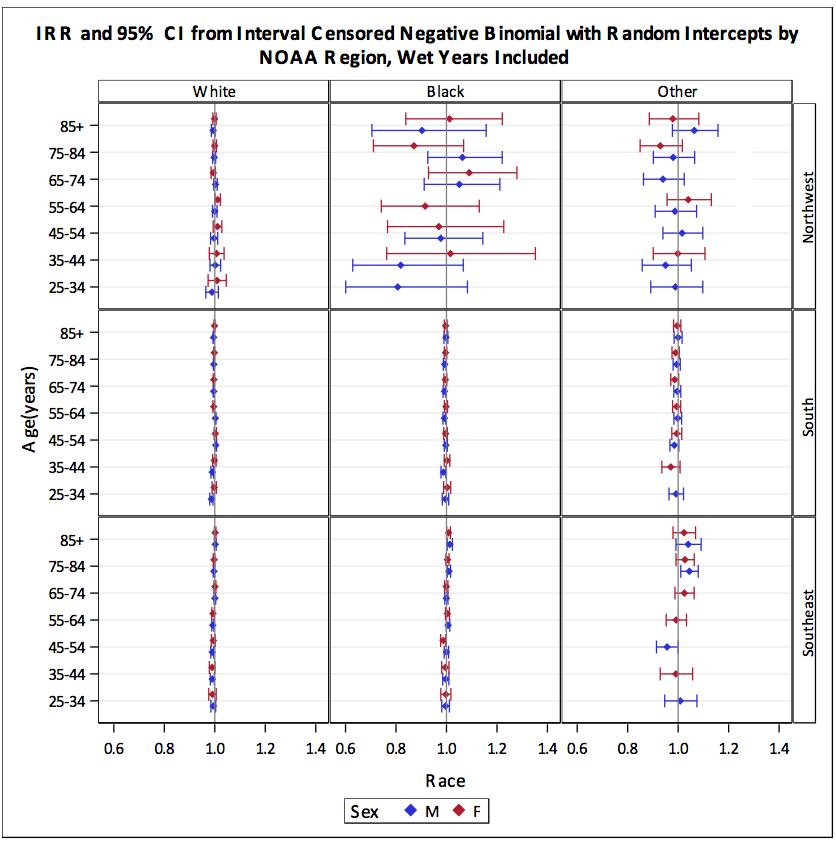

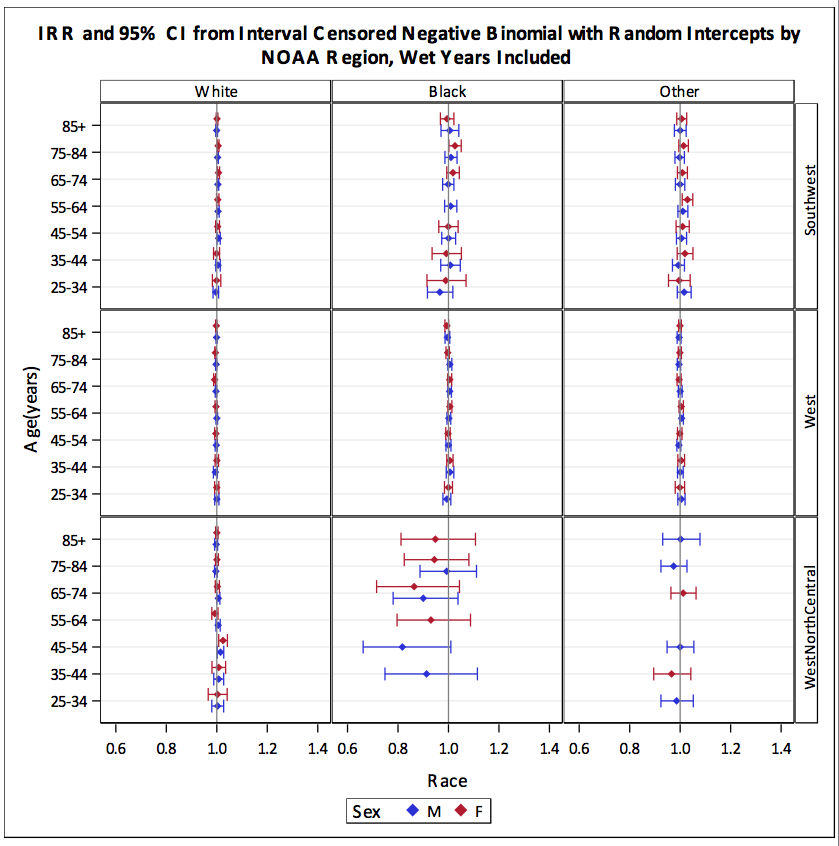

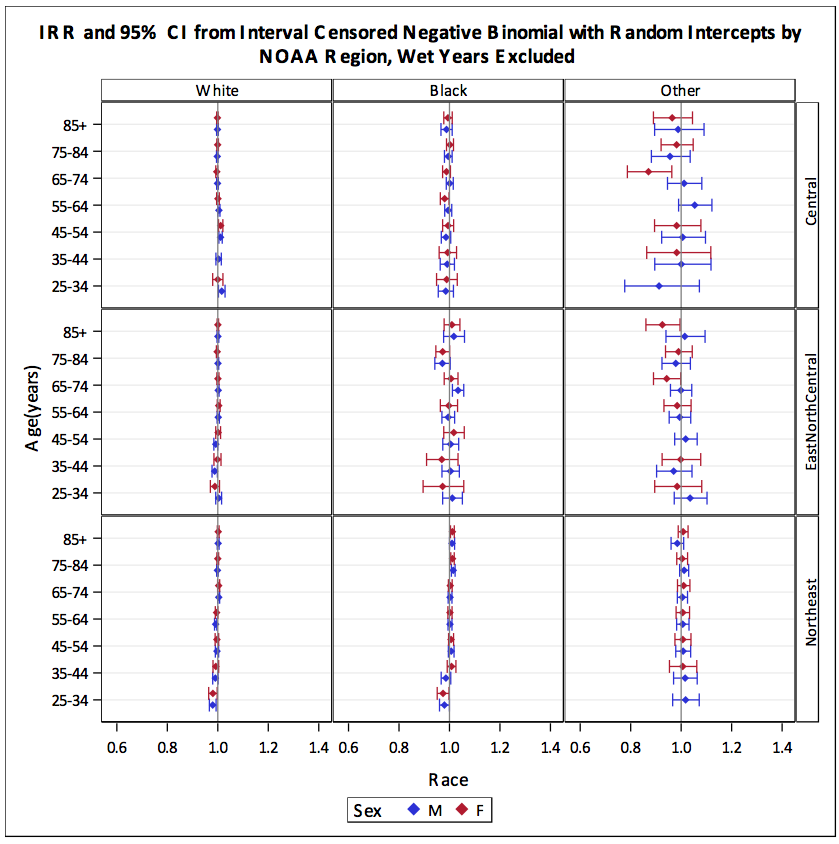

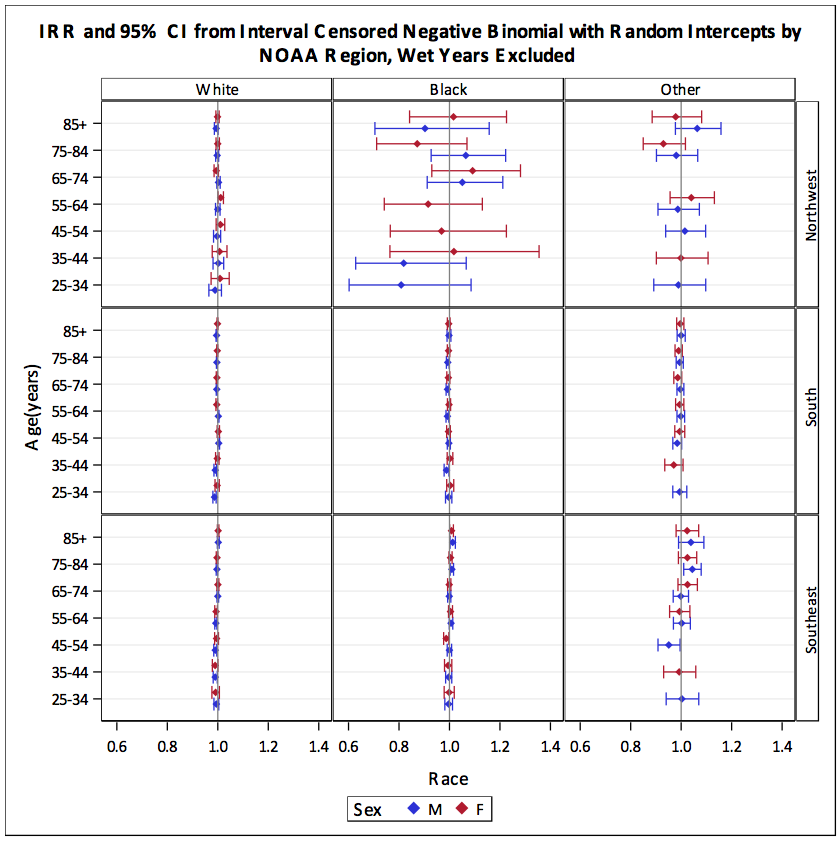

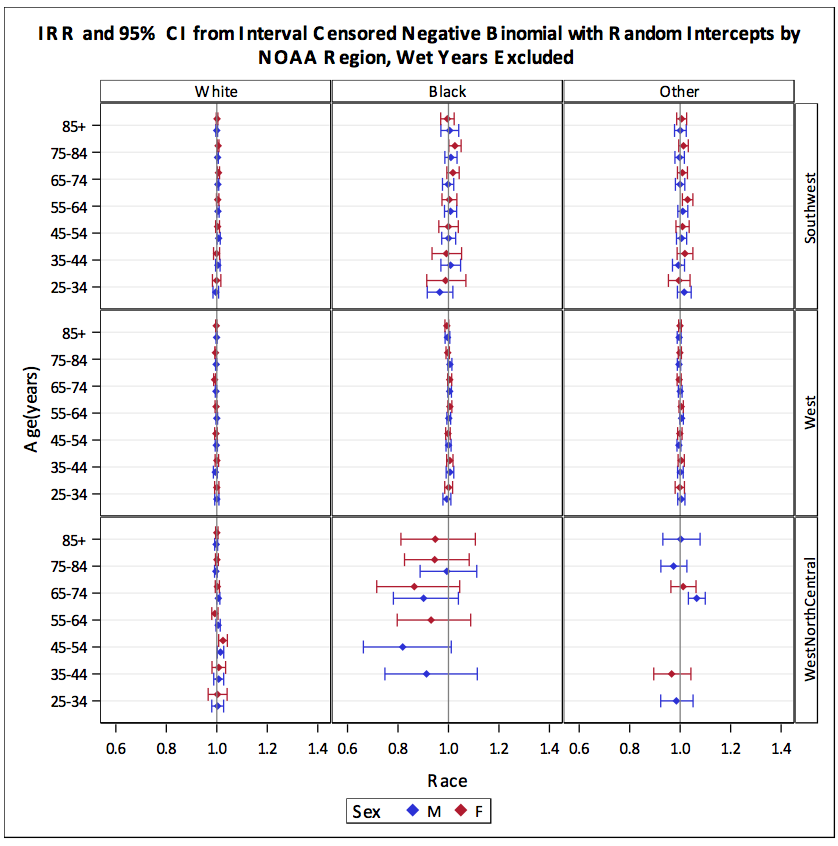

Supplement: Supplementary file 4 — Additional file 4: Appendix IV. Forest Plots [file 12940_2020_597_MOESM4_ESM.docx]
